# Analysis of trend of malaria prevalence in south-west Ethiopia: a retrospective comparative study

**DOI:** 10.1186/1475-2875-13-188

**Published:** 2014-05-24

**Authors:** Lelisa D Sena, Wakgari A Deressa, Ahmed A Ali

**Affiliations:** 1 Department of Epidemiology, College of Public Health and Medical Sciences, Jimma University, Jimma, Ethiopia; 2 Department of Preventive Medicine, School of Public Health, College of Health Sciences, Addis Ababa University, Addis Ababa, Ethiopia

**Keywords:** Ethiopia, Gilgel-Gibe, Health service, Malaria trend, Malaria prevalence

## Abstract

**Background:**

The temporal analysis of pertinent malaria data on the health care system is crucially important to measure success or failure of malaria programmes and identify remaining malaria hot spots. The objectives of this study were to analyse and compare trends of malaria prevalence around Gilgel-Gibe Hydroelectric Dam (GGHD), and a control site over an eight-year period.

**Methods:**

A retrospective record review of health care services was conducted in southwest Ethiopia. Records of malaria cases over an eight-year period in primary health care units of two localities were reviewed. One study site was selected from villages around a man-made lake, GGHD, within a distance of 10 km, and a control site with similar geographic features was identified. Data were summarized in tables; prevalence of malaria was analysed and described by person, place and time using line graphs. Odds ratio was used to examine significant difference of malaria occurrence in the two sites.

**Results:**

Records of 163,918 malaria cases registered over eight years (September 2003 to August 2011) were explored. Close to one thirds (32.7%) of these cases were from GGHD site and two-thirds (67.3%) of them were from the control site. Among the confirmed cases, *Plasmodium falciparum* constituted 54.6%, *Plasmodium vivax* accounted for 41.6%, and mixed infection was 3.8%. There were three peaks of malaria prevalence in the control site whereas only one major peak was identified during the eight-year period in GGHD site; and prevalence of malaria in GGHD site was lower than control site. Children in the age range ten to 14 years were the most affected by the disease, followed by children below the age group five to nine years, which demands due consideration in the effort of malaria control.

**Conclusions:**

More malaria prevalence was observed in the control site compared to GGHD site almost throughout the time period considered. The present finding did not show evidence of the excess malaria burden in the GGHD site due to the presence of the dam.

## Background

Malaria has been a major public health problem throughout human history, particularly in the tropical and subtropical parts of the world [1]. In Ethiopia, the occurrences of epidemics of malaria have been documented since the 1930s and 1940s. The most devastating epidemic was that of 1958 which resulted in an estimated three million cases and 150,000 deaths [2,3]. Until recently, malaria has been the leading cause of morbidity and mortality in Ethiopia [4-6]. The two peak seasonal transmissions of malaria occur during the months of September to December and March to May [4,7].

Studies have shown that the *Plasmodium* species compositions and the number of malaria cases vary over time [8-11] due to different factors, such as previous weather conditions [12] or intervention measures [13]. A hospital-based retrospective study done in Ethiopia revealed decrement of *Plasmodium vivax* whereas *Plasmodium falciparum* increased over a five-year period [7]. Another ten-year trend study carried out in Jimma Town, Ethiopia, showed not only fluctuation of number of malaria cases but also changes in the species of *Plasmodium* composition following climatic variables [14].

Understanding how malaria varies in the community as a result of seasonal or year-to-year changes is essential for planning national malaria control programmes [15]. The temporal analysis of relevant malaria data of health care system gives essential information needed to measure achievements of national malaria programmes and scrutinize remaining malaria hot spots. It also gives important insight into the changing malaria situation, which might guide adjustments of malaria programme activities and the prioritization of malaria research [16,17]. Therefore, the changing malaria situation requires an updating description of malaria trends [18].

Comprehensive studies regarding prevalence of malaria over a period of time that includes all age segments of the population, taking into account the base population (denominator), are scarce in Ethiopia in spite of the recognition of the disease burden for more than half of a century. Therefore, the objectives of this study were to analyse and compare trends of malaria prevalence around Gilgel-Gibe hydroelectric dam (GGHD) and a control site and to identify trends of *Plasmodium* species over the time-period considered.

## Methods

### Study settings

This record review comparative study was conducted in two rural settings; one site was surrounding a man-made lake, GGHD, which started operating in 2004 [19], and a control site was more than ten km far away from the lake so that the possible effect of the dam would not be reflected in the control site. The GGHD site is a health and demographic surveillance site (HDSS) [20] which includes surrounding villages within ten km radius of the GGHD and about 50,000 residents used to live in the villages. The control site was selected considering the similarity of geographic features with the Dam site regarding altitude and presence of rivers, but without dam, and equivalent ranges of distance from water bodies as main risk of malaria. The population of the two sites had similar socio-demographic characteristics and economic activities. Both sites are found within altitude range which favors seasonal malaria transmission [21-23]. A previous survey showed that the two sites had equivalent household sizes of about five individuals per household [24], thus, equivalent villages and small towns with that of the GGHD were identified from the control site with about 50,000 mid-year population; thus, an estimated 100,000 mid-year population contributed to the malaria cases from the two study sites.

The residents of both study sites had accessibility to primary health care units. A primary health care unit consists of a health centre and, on average, five health posts together serving about 25,000 population in a radius of about five km of the health centres. The health centres are usually staffed by middle level health professionals, including laboratory technicians and technologists, thus cases with fever are often tested for malaria. The health posts are staffed by health extension workers as of 2005 [25]; and they could perform rapid diagnostic tests for malaria suspected cases and treat uncomplicated malaria cases. Detailed descriptions of the study settings have also been given elsewhere [21,22,24].

### Study design

A retrospective comparative study design was employed using data from local health services (health centres and health posts). This was done by reviewing malaria morbidity records of local health facilities pertaining to villages within a specified radius of the GGHD and the control site. Trend of malaria prevalence was analysed and compared by person and place.

### Source of data and sample size

In Ethiopia, malaria cases are treated both clinically and as confirmed malaria accordance to national guideline [6,26] depending on the degree of diagnostic capabilities at different levels of the healthcare system. Both the presumptive and confirmed cases are registered on preformatted registration books (log books) at health care levels and reported both weekly as well as monthly to next higher level of health management system. The present study included all malaria records of the local primary healthcare units that were within the environs of GGHD (Gilgel Gibe DHSS) and control villages registered by the primary health care units between September 2003 and August 2011 were reviewed. In the GGHD site there were eight health posts and three health centres from where records of malaria cases visited the health services only from villages included into the Gilgel Gibe DHSS were considered for the present study. Similarly, villages in the control site had eight health post and one health centre from where only malaria records of the selected villages included into this study. The timeline consideration was based on data availability both for GGHD as well as control sites; and the fact that significant change in the number of malaria cases, such as epidemics, occurs in cyclic fashion from five to eight-year periods in the country [4,27,28], in order that any fluctuation of such cycle could be captured.

### Data collection techniques

A format was prepared on a computer spreadsheet (Excel) to collect the secondary data from log books of local government primary health care units of the area. Individual level data on malaria morbidity; such as, diagnosis results (negative, species of *Plasmodium* for positives); dates of diagnoses; available demographic data (residence, age, sex) were registered on the computer spreadsheet. All records of patients who visited the health institutions during the timeframe considered and treated as malaria patients were included in the study. Records of cases with incomplete records, such as dates of health service visit, age, address, results of diagnosis, were excluded from the analysis.

### Data management and processing

The data on malaria case records of different local healthcare institutions of the study sites were transferred to Excel spreadsheet, checked for completeness (date of diagnosis, test results, patient age and address), coded and combined on the same spreadsheet; then, prepared data of malaria cases were exported to SPSS statistical software version 20 for Windows. Subsequent analyses were performed using the SPSS statistical software. Results were summarized using tables and figures and odds ratios were used to estimate the level of difference between the study sites in the distribution of malaria prevalence.

### Ethical considerations

Ethical clearances were obtained from the Addis Ababa University College of Health Sciences Ethical Review Board and Oromia Regional Health Bureau Ethical Clearance Committee. Jimma Zonal and District Health offices as well as local administrators were communicated by formal letters written from School of Public Health of Addis Ababa University and Oromia Regional Health Bureau. Individual information was kept confidential and the names of individuals were removed from data of health services and identified only by identification numbers and the results were communicated in an aggregated manner.

## Results

This retrospective study examined records of 163,918 malaria cases registered over eight years (September 2003 to August 2011). Close to a third (32.7%) of those cases were from the GGHD site and two-thirds (67.3%) were from the control site. Overall, the male to female ratio was 1.08: 1 and slightly higher for the GGHD site (1.12:1 *vs* 1.06: 1) than the control site. The under-five age group constituted 12.0% of all the cases, which was higher in the control site (12.7% *vs* 10.5%) than GGHD site. The proportion of children aged from five to fourteen years was 28.0% (28.3% in control *vs* 27.6% in GGHD); the proportion in the age group ten to fourteen years was higher among the control (16.3% *vs* 14.4%) than the GGHD site. On the other hand, the composition of adult cases aged 15 years and above was higher in the GGHD site (62.3% *vs* 59.0%) than the control site. Regarding the composition of age group between males and females within the study sites, there was similarity in the GGHD site whereas in the control site more girls in the age range ten to fourteen years were affected (17.2 *vs* 15.3%) than counterpart boys; however, in the adult age category more males than females (60.7 *vs* 57.1%) were infected (Table 1).

**Table 1:** Distribution of malaria cases by sex, age and study sites, southwest Ethiopia, September 2003-August 2011

**Sex**	**Age group in years**	**Gilgel-Gibe**		**Control**		**Total**
		**N (%)**	**Row %**	**N (%)**	**Row %**	**N (%)**
Male	<5	2,954 (10.4)	28.5	7,220 (12.7)	71.5	10,174 (12.0)
	5-9	3,540 (12.5)	38.8	6,353 (11.2)	61.2	9,893 (11.6)
	10-14	4,015 (14.2)	36.3	8,712 (15.3)	63.7	12,727 (15.0)
	15+	17,829 (62.9)	33.3	34,480 (60.7)	66.7	52,309 (61.5)
	**Subtotal**	**28,338 (100.0)**	**33.3**	**56,765 (100.0)**	**66.7**	**85,103 (100.0)**
Female	<5	2,659 (10.5)	28.9	6,801 (12.7)	71.1	9,460 (12.0)
	5-9	3,320 (13.1)	35.2	6,871 (12.8)	64.8	10,191 (12.9)
	10-14	3,733 (14.8)	32.6	9,273 (17.3)	67.4	13,006 (16.5)
	15+	15,574 (61.6)	32.3	30,584 (57.1)	67.7	46,158 (58.6)
	**Subtotal**	**25,286 (100.0)**	**32.1**	**53,529 (100.0)**	**67.9**	**78,815 (100.0)**
Total	<5	5,613 (10.5)	28.7	14,021 (12.7)	71.3	19,634 (12.0)
	5-9	6,860 (12.8)	37.0	13,224 (12.0)	63.0	20,084 (12.3)
	10-14	7,748 (14.4)	34.4	17,985 (16.3)	65.6	25,733 (15.7)
	15+	33,403 (62.3)	32.8	65,064 (59.0)	67.2	98,467 (60.1)
	**Total**	**53,624 (100.0)**	**32.7**	**110,294 (100.0)**	**67.3**	**163,918 (100.0)**

About two-thirds were confirmed uncomplicated malaria cases and a third were clinical malaria cases, including probable malaria cases. Overall, *P. falciparum* and *P. vivax* accounted for one-third and one-quarter of all the cases, respectively, with similar proportion across all age groups. Pertaining to the species composition among the confirmed cases, *P. falciparum* constituted 54.6% (60.4% in GGHD site and 52.3% in the control site), *P. vivax* accounted for 41.6% (33.6% in GGHD and 44.7% in control) and mixed species was 3.8% (6.0% in GGHD and 3.0% in control) of the confirmed cases.

There have been higher proportion of infections in control site by both *P. falciparium and P. vivax* species in all age categories. Infection by *P. falciparum* and *P. vivax* malaria was 1.5 and 2.2 times more likely to occur in the control site compared to GGHD site among the under five years of age group. The prevalences of *P. falciparum* and *P. vivax* was respectively more than two and three times likely to occur among children five to fourteen years age group in the control sites compared to the GGHD site. Among the adults fifteen years and above years of age, the prevalences of *P. falciparum* and *P. vivax* were 1.7 ans 2.7 repectively more likely to happen in the control sites compared to the GGHD site (Table 2).

**Table 2:** Distribution of confirmed malaria cases by age group and study sites, southwest Ethiopia, September 2003-August 2011

**Age group (year)**	**Diagnosis**	**GGHD**	**Control**	**Odds ratio (C.I.)**
		**N (%)**	**N (%)**	
<5	*P. falciparum*	1437 (23.8)	4606 (76.2)	0.65 (0.53,0.80)*
	*P. vivax*	901 (18.2)	4056 (81.8)	0.46 (0.38,0.57)*
	*P. mixed*	145 (32.4)	302 (67.6)	1
	**Sub total**	**2483 (21.7)**	**8964 (78.3)**	
5-9	*P. falciparum*	2293 (33.6)	4530 (66.4)	0.47 (0.39,0.56)*
	*P. vivax*	1239 (23.9)	3954(76.1)	0.29 (0.24,0.35)*
	*P. mixed*	261 (52.1)	240(47.9)	1
	**Sub total**	**3793(30.3)**	**8724 (69.7)**	
10-14	*P. falciparum*	2260 (27.2)	6053 (72.8)	0.45 (0.38,0.54)*
	*P. vivax*	1323 (20.5)	5143 (79.5)	0.31 (0.26,37)*
	*P. mixed*	263 (45.1)	320 (54.9)	1
	**Sub total**	**3846 (25.0)**	**11516 (75.0)**	
15+	*P. falciparum*	10939 (33.2)	22045 (66.8)	0.61 (0.57,0.67)*
	*P. vivax*	5947 (24.2)	18676 (75.8)	0.40 (0.36, 0.43)*
	*P. mixed*	1011 (44.5)	1262 (55.5)	1
	**Sub total**	**17897 (29.9)**	**41983 (70.1)**	
Total	*P. falciparum*	16929 (31.3)	37234 (68.7)	0.58 (0.54,0.61)*
	*P. vivax*	9410 (22.8)	31829 (77.2)	0.37(035,0.40)*
	*P. mixed*	1680 (44.2)	2124 (55.8)	1
	**Total**	**28019 (28.2)**	**71187 (71.8)**	

*Significant at P = 0.0001.

**Figure 1:**
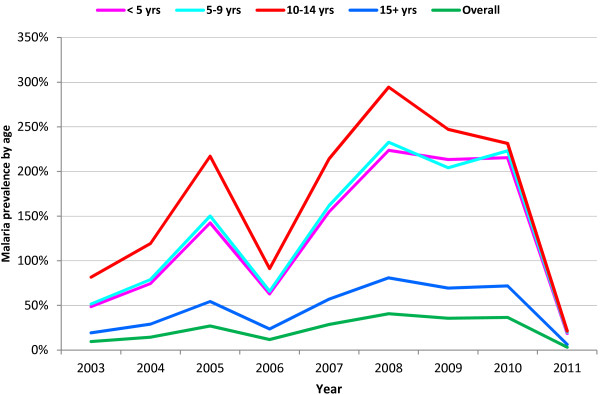
Trend of confirmed malaria prevalence by age group and year, southwest Ethiopia, September 2003 - August, 2011.

There was variation of trend of confirmed malaria among different age groups as well as year to year trends. Children in the age range from ten to fourteen years were the most affected by the disease. As depicted in Figure 1 (red line), the prevalence of malaria was highest in this age category from 2003 to 2010 when the prevalence of malaria dropped in all age groups. Especially, in 2005, a child in this group got more than two infections and, on average, a child got nearly three infections in 2008. Children below the age of ten years were the second most affected following those aged ten to fourteen years. In 2005, children below ten years got on average 1.5 infections and from 2008 to 2010, they got more than two infections of *Plasmodium* yearly. However, there was not much pronounced risk difference between children in the age group below five years (pink line) and those five to nine years (cyan line) age categories, although the latter was slightly higher.On the other hand, the adult age group (blue line) was the least affected. In this age category, the prevalence of malaria reached 50% in 2005 and decreased by half the following year. However, the prevalence gradually increased and reached about 75% in 2008 where it levelled for the subsequent two years. The green line in the figure shows the overall prevalence of confirmed malaria in all age groups, which is seemingly lowest of all obscuring the burden of the disease in different age categories particularly in the children categories (Figure 1).

There was also a difference in the pattern of malaria prevalence between the study sites. Generally there were three peaks of malaria prevalence during the eight-year period considered in the control site. The three significant malaria prevalence peak periods were in 2005, 2008 and 2010. In 2005, the prevalence of malaria reached 25% and the other two peaks were above this figure. The proportion of *P. falciparum* (brown line) and *P. vivax* (blue line) was almost equivalent throughout the period considered irrespective of the overall variation over time. The *P. falciparum* prevalence was slightly higher than that of *P. vivax* except in 2008 and 2010 when the *P. vivax* prevalence was elevated a little above *P. falciparum*.

**Figure 2:**
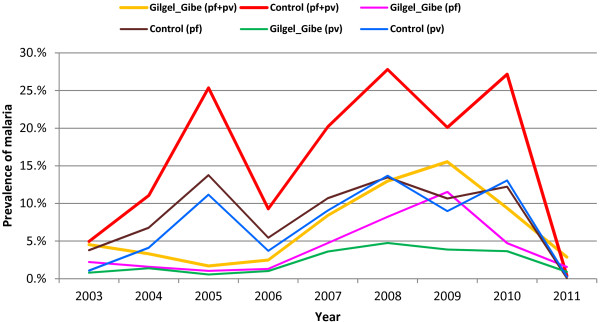
Trend of confirmed malaria prevalence by study sites, species composition and year, southwest Ethiopia, September 2003 - August 2011.

On the other hand, prevalence of malaria in GGHD (orange line) was much lower than in control site (red line) and exhibited only one major peak during the eight-year period. In the GGHD site, malaria prevalence was low and showed a slight decline before 2006; and the composition of *P. falciparum* (pink line) was only slightly higher than *P. vivax* (green line) species. Since 2006 however, the prevalence of malaria steadily increased in the site. The *P. falciparum* infection was increased excessively and reached its peak in 2009 and declined the following two years. The increment of *P. vivax* prevalence was more gradual and it reached its highest level in 2008 and showed a very gradual decline since then (Figure 2).

**Figure 3:**
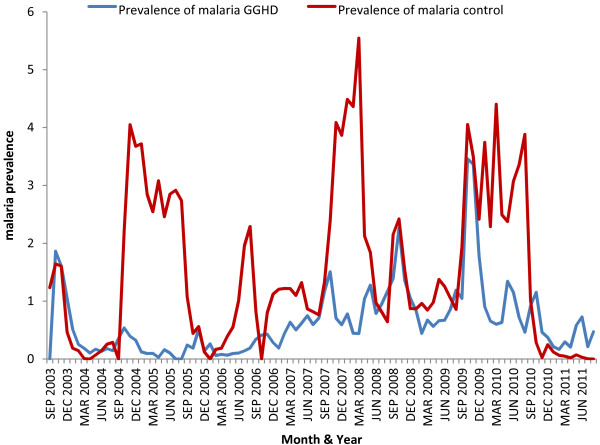
Trend of confirmed malaria prevalence by study sites and quarters of year, south-west Ethiopia, September 2003-August 2011.

Malaria prevalence was decreased both in GGHD and control sites from September 2003 to August 2004, then the patterns of malaria prevalence in the sites gradually followed different patterns. In the control site, the prevalence of malaria had been excessively higher since September 2004. From September 2004 onwards, the malaria prevalence was remained at higher levels throughout the year and dropped sharply in September 2005 and further reduced in December 2005. From March to September 2006 there was a peak during the six months. From September 2007 to July 2008, the highest rise of malaria prevalence was noticed in the control site. There were still peaks of malaria prevalence evident in the area during September to December 2008 and September 2009 to September 2010 for about two subsequent years until it dropped drastically almost to zero level in September 2011.In the GGHD area, malaria prevalence was low starting from December 2003 to September 2006 with two small rises during the season of September to November 2004 and 2005. However, there was persistent and gradual increment of malaria prevalence during the subsequent four years with peak transmission of different magnitude during the months of September 2007, 2008 and 2009. The peak in 2008 coincided with that of the control area peak and the rise of prevalence in September 2009 was the highest one in the GGHD area. Since March 2010, malaria prevalence has decreased steadily in the GGHD area as well (Figure 3).

**Figure 4:**
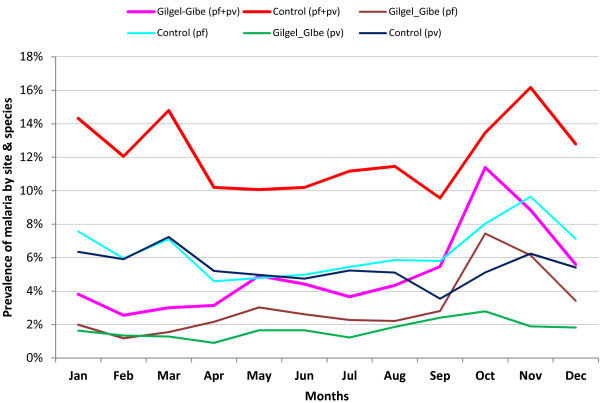
Trend of confirmed malaria prevalence by study sites, species and month, southwest Ethiopia, September 2003 - August 2011.

The average monthly distribution of malaria prevalence exhibited different patterns between the study sites. Generally, two major peaks were noticed in the control site (red line). One peak was during the start of rainfall February to May and the other one was after the heavy rainfall from September up to December. The contribution of *P. falciparum* (cyan line) and *P. vivax* (navy line) was almost the same during the first wave but the *P. falciparum* contribution was found to be higher during the September to November peak transmission in general. In contrast, the prevalence of malaria in GGHD site (pink line) showed one major peak which was from September to November and of relatively shorter duration. The increment of malaria prevalence in GGHD site was entirely due to *P. falciparum* (brown line) infection which occurs usually in the form of epidemics. The contribution of *P. vivax* (green line) was very low and almost constant throughout the years (Figure 4).

## Discussion

The essence of this study was to estimate and compare the prevalence of malaria, identify possible shift of *Plasmodium* species compositions over time in the two localities from the available data of the health services. The study considered only key individual characteristics (age and sex), date of diagnosis, diagnosis outcome and address of the cases in an attempt to minimize the usual limitations of secondary data such as incompleteness of information. In the country, mostly malaria cases are diagnosed and treated at primary health care level. Health extention workers who had been trained and deployed at village level since 2005 [25] can conduct also rapid diagnostic tests for malaria and treat uncomplicated cases at health posts level which are part of the primary health care unit. Such expansion of health services at grass root level has increased accessibility to health services and hence, there has been reports of positive health outcomes [29,30]. Yet, the two study sites have been exposed to similar improvement in health delivery system and such changes would not compromise the comparability of distribution of malaria in the two sites. Moreover, as the study dealt with a relatively large set of data, the appearance of findings due to chance is expected to be minimized. Thus, this retrospective healthcare service-based study is believed to have assessed the distribution of malaria over a period of eight years by person, place, time, and composition of *Plasmodium* species.

The prevalence of malaria was excessively higher in the control site and characterized by several peaks of transmission through the timeline considered. Irrespective of the scale-up of long-lasting, insecticide-treated bed nets (LLINs) since 2005 [5], the number of cases steadily increased from 2006 to 2008 when it reached the highest level in both study sites. Especially, *P. falciparum* malaria showed a drastic rise in the GGHD site during that period, possibly due to resistance development by malaria vector mosquitoes to DDT (75% WP) and partly Malathion (50% WP) that was in use for indoor residual spraying (IRS) in the country since 1960s until malaria vector resistance to DDT was detected in 2007 [31-33]. The increment of malaria cases in GGHD site was still by far below that of the control site despite of the earlier report of a study [22], which was conducted a year after the construction of the dam in 2004, that indicated higher risk of malaria infection among children who lived within three kilometer radius of the GGHD as compared to those who lived eight to ten kilometer away from the dam. Again, a more recent report of longitudinal study of the same area showed that there was no significant malaria risk difference between children who lived in villages near to the dam and those who lived away from it and malaria was reported to be highly seasonal driven in the area [23]. This implies that access malaria risk due to the dam construction was not clearly evident over the long period of time as far as interventions are in place like other malarious areas.

After 2009 in the GGHD and 2010 in the control sites, sharp decline of the number of malaria cases was noticed, possibly due to the replacement of old LLINs in 2009, followed by shifting of insecticide used for IRS in 2010 to deltamethrin or bendiocarb insecticides [31], such coincidence of interventions followed by a drastic drop of malaria was also revealed by other findings [17,34-37] in other African countries. This suggests the need for the effective combined intervention methods using IRS with potent insecticides and LLINs as evidenced by different studies [38-40], as well as the need to monitor insecticide resistance of mosquitoes.

The contribution of *P. falciparum* was higher (64.3% *vs* 53.9%) in GGHD site than control site. This proportion of *P. falciparum* in GGHD site was similar to health facility-based studies in Ethiopia [14,21], but less than other local findings [5,41,42] and still by far lower than other local findings of community based surveys [39,43]; however, the proportion of *P. falciparum* in the control area lower than the report of health facility based study findings in Ethiopia. The proportion of *P. vivax* malaria was above 40% except in 2003/4 and 2008/9 when it was 25.1% and 37.9%, respectively. Especially, the proportion of *P. vivax* was higher (44.4% *vs* 33.6%) in the control site which might have contributed to the higher level of malaria prevalence in the site as it occurred almost at the higher and stable level during the time period considered.

The present study shows that slightly more males were affected than females which are similar to other findings [41,44-47] and, all age groups were found to be susceptibe to malaria. However, children were more affected than adults, especially those in the age range ten to fourteen years were disproportionally affected. Similarly, other studies have reported susceptibility difference that children were more affected [48,49] than older age groups, but other studies have reported more susceptibility in adult age groups [46,50]; the latter might be confounded by other factors.

Reporting by absolute number or proportion of cases can be misleading unless the denominator of each age category is considered. For instance, in this finding Table 1 shows that the proportion of adult cases is higher by many fold than that of children; however, consideration prevalence (Figure 1), which takes into account respective denominators, points out that the children were actually the most affected ones. The fact that children aged from ten to fourteen years were more affected than those below ten years suggests not susceptibility difference but exposure difference, such as giving priority to small children in the use of LLINs. This implies that analysis of extent of malaria burden needs to take into consideration the base (denominator) population.

In this study, the use of secondary data might have underestimated the extent of the malaria burden in both sites. However, this condition would not introduce systematic bias in the analysis and comparison of trends of malaria prevalence, as both sites might have a similar chance of missing/capturing malaria records. Again, malaria treatment in government health care services is for free in Ethiopia, so that most of the malaria cases, if not all, are expected to be more attracted to local government health care services than to private health institutions, which are on payment basis. Therefore, this study is believed to have reflected the true picture of malaria burden and its trend in the study sites.

## Conclusions

Malaria occurrence appeared to follow different patterns in the two study sites. Two seasonal peak transmissions were annually observed in the control site due to both *P. falciparum* and *P. vivax* infections. However, only one short seasonal transmission was noticed in the GGHD site due to *P. falciparum* while transmission of *P. vivax* infection was at low and constant level throughout the considered period. The short duration rise of *P. falciparum* malaria in GGHD implies that malaria was driven by seasonal influence but not due to effect of the dam which would otherwise indicated persistent transmission of longer duration of the disease.

All age groups of the population were affected by malaria; however, the fact that children in the age group five to fourteen years were more affected by the disease suggests not higher susceptibility level but higher exposure status due to less attention given to these older children than the under five years of age. Therefore, due attention needs to be given to this age category together with the ‘vulnerable’ segments of the population.

## Abbreviations

GGHD: Gilgel-gibe hydroelectric dam; LLINs: Long-lasting insecticide-treated bed nets; OR: Odds ratio; SPSS: Statistical package for social sciences; Vs: Versus; WHO: World Health Organization; WP: Wetable powder.

## Competing interests

The authors declare that they have no competing interests.

## Authors’ contributions

LDS participated in the study design, undertook the field study, analysed data and wrote the manuscript. WAD participated in the study design, revision of the manuscript and facilitation of administrative issues. AAA participated in the study design, revision of the manuscript and facilitation of administrative issues. All authors have read the manuscript and approved it to submit for publication.
